# Reflections on so-called culture-negative osteoarticular infections in pediatric populations

**DOI:** 10.3389/fped.2026.1775415

**Published:** 2026-05-11

**Authors:** Ahmer Ahmad Khan, Giacomo De Marco, Elio Paris, Oscar Vazquez, Christina Steiger-Tuk, Romain Dayer, Dimitri Ceroni

**Affiliations:** 1 Pediatric Orthopedics and Traumatology Unit, Department of Pediatric Surgery, Geneva University Hospitals, Geneva, Switzerland; 2 Faculty of Medicine, University of Geneva, Geneva, Switzerland

**Keywords:** culture-negative infections, fastidious microorganisms, HACEK organisms, Kingella kingae, next-generation sequencing, oropharyngeal microbiota, pediatric osteoarticular infections, subacute hematogenous osteomyelitis

## Abstract

Osteoarticular infections (OAIs) in children persist to be a major clinical concern due to their potential long-term impact on bone growth and joint function. Despite careful sampling, a significant proportion of OAIs remain culture-negative, historically obscuring the true epidemiology of these conditions. At the same time, subacute osteomyelitis is increasingly being detected and recognized as a distinct osteoarticular infection. Subacute hematogenous osteomyelitis (SAHO) is often under-recognized, as it is typically characterized by localized pain, mild or absent systemic manifestations, non-contributory laboratory tests and blood cultures, but positive radiological findings. This review focuses on the phenomenon of so-called culture-negative OAIs, exploring their microbiological basis, the role of fastidious and atypical microorganisms, parallels with subacute osteomyelitis, and the role of modern diagnostic technologies.

## 1 Introduction

Osteoarticular infections (OAIs) remain a major pediatric problem with potential long-term effects on joint function and bone growth (1). Identifying the causative pathogen is crucial for diagnostic accuracy, appropriate antibiotic selection (2), and improved outcomes (3). Historically, a limited panel of pathogens was recognized, with *Staphylococcus aureus* considered the predominant cause of pediatric OAIs (4–9). This simplified view largely reflected the limitations of classical culture-based methods. Indeed, 24%–68% of acute hematogenous osteomyelitis (AHO) and 21%–70% of septic arthritis (SA) cases remain culture-negative despite rigorous sampling (10, 11). This diagnostic gap has clearly biased our understanding of the bacteriologic etiology and epidemiology of pediatric OAIs.

Our understanding of the microbiology and clinicobiological features of OAIs has advanced substantially in recent years. New diagnostic methods include immunoassays that detect microbial antigens and nucleic acid amplification tests (NAATs). Since the early 2000s, NAATs capable of detecting very small quantities of bacterial nucleic acids in clinical samples have improved in sensitivity and specificity (12, 13). Their introduction into clinical laboratories has markedly improved OAI diagnosis and reshaped contemporary bacteriologic epidemiology. Recent work has shown that *Kingella kingae* is now the most common pathogen in children under 4 years of age (14–17), underscoring the value of species-specific real-time quantitative PCR (qPCR) assays.

Despite wide adoption of PCR (14, 16, 17), more than 20% of OAI cases still lack an identified pathogen. This suggests that a substantial proportion of culture-negative OAIs are due to yet-unrecognized fastidious organisms for which validated primers are unavailable, highlighting the need for further characterization (14, 16, 17). The next few years should focus on culture-negative OAIs to identify additional causative organisms. When targets will be selective sequencing of specific genomic regions could accelerate pathogen identification while reducing overall time to diagnosis (18).

## 2 Epidemiology of culture-negative OAIs

Culture-negative OAIs represent a substantial proportion of pediatric osteoarticular infections. Reported rates vary widely by study design, sampling strategy, and diagnostic technique. In acute hematogenous osteomyelitis, culture-negative cases account for 24%–68% of presentations, while in septic arthritis this proportion ranges from 21 to 70% (10, 11). These figures highlight a persistent diagnostic gap that has profound implications for antibiotic stewardship and epidemiological surveillance.

The introduction of PCR-based assays has significantly narrowed this gap. Several studies have demonstrated a two- to threefold increase in pathogen identification rates when molecular methods are used alongside or in place of conventional culture. In particular, *K. kingae*, a fastidious Gram-negative organism, was largely undetected before the advent of PCR, yet it is now recognized as the leading cause of OAI in children under 4 years of age in Switzerland and Western Europe (14–17). This shift in the bacteriologic landscape underscores that many historical culture-negative cases were, in fact, caused by identifiable organisms that simply escaped conventional detection.

Subacute presentations add another layer of complexity to the epidemiology of culture-negative OAIs. Subacute hematogenous osteomyelitis (SAHO), defined by its indolent course, minimal systemic signs, and positive radiological findings in the absence of microbiological confirmation, represents a distinct but overlapping entity. Data from Juchler et al. and Coulin et al. suggest that PCR positivity in SAHO exceeds 60%, compared to less than 25% for culture (14, 17). This reinforces the importance of molecular diagnostics in this setting (Table 1). More recently, the largest single-center retrospective series on SAHO, including 107 consecutive cases over 25 years (Paris et al., 2026), confirmed that conventional culture alone identified a pathogen in only 17.8% of cases, while combined use of culture and multiple PCR strategies raised this figure to 44.9% (19). Notably, 20 additional patients tested positive for *K. kingae* DNA on oropharyngeal swab alone, despite negative blood cultures and bone sampling, highlighting the non-invasive diagnostic potential of throat swab PCR in this population.

**Table 1:** Comparison of culture vs. PCR detection rates in pediatric OAIs and SAHO.

Study	Population	Culture Yield	PCR Yield	Key Finding
Chometon et al.	Pediatric OAI	30 to 40%	70 to 80%	*K. kingae* identified in majority of PCR-positive cases
Peltola et al.	Pediatric septic arthritis	45%	Not reported	Culture-negative cases with favorable outcomes suggesting fastidious organisms
Juchler et al. (14)	SAHO	<20%	>60%	PCR identified *K. kingae* in a substantial proportion of PSAHO cases
Coulin et al. (17)	SAHO	<25%	65%	Molecular diagnosis superior in subacute presentations
Samara et al. (16)	Pediatric OAI	35%	68%	NGS identified unexpected pathogens in culture-negative cases
Paris et al. (19)	PSAHO (*n* = 107)	17.8%	44.9%	Combined culture and PCR identified pathogen in 44.9% of cases; *K. kingae* throat swab PCR positive in 20 additional cases

SAHO, Subacute Hematogenous Osteomyelitis; PSAHO, Primary Subacute Hematogenous Osteomyelitis; OAI, osteoarticular infection; PCR, polymerase chain reaction; NGS, next-generation sequencing.

## 3 Microbiological basis: fastidious organisms

### 3.1 The oropharyngeal microbiota as the main source of OAIs in children

In children, there is now a general agreement that most pathogens responsible for primary OAIs are derived from the oropharyngeal microbiota. The microbiota resides on or within several human tissues and biofluids, including the skin, the gastrointestinal tract, and the oropharyngeal mucosa (20). Commensal bacteria colonize the host shortly after birth and gradually develop into highly complex ecosystems during growth (21). The human body harbors thousands of bacterial species and carries a microbial gene repertoire approximately 150 times larger than that of the human genome (20, 22, 23).

The oral cavity and associated nasopharyngeal region provide an ideal environment for microbial growth. The mean normal oral-pharyngeal temperature is 37 °C, offering a stable habitat, while saliva provides a pH between 6.5 and 7.5 and acts as a nutrient medium (24). The oral cavity harbors more than 700 bacterial species, making it one of the most densely populated anatomical sites in the human body (24).

In healthy children, the oropharyngeal microbiota is composed primarily of *Prevotella*, *Capnocytophaga*, *Campylobacter*, *Veillonella*, *Streptococcus*, *Neisseria*, and *Haemophilus*. Many pathogenic species, including *Streptococcus pneumoniae*, *Streptococcus pyogenes*, *Staphylococcus aureus*, *Haemophilus influenzae*, *Neisseria meningitidis*, and *Mycoplasma pneumoniae*, are well-adapted to the pharyngeal environment and may colonize the host asymptomatically. Additionally, HACEK organisms (*Haemophilus*, *Aggregatibacter*, *Cardiobacterium*, *Eikenella*, *Kingella* spp.) are part of this microbiota and represent an important reservoir of fastidious pathogens.

Maintaining microbiota homeostasis helps prevent disease: commensals exert competitive pressure on potential pathogens, and disruption of this balance, known as dysbiosis, can predispose to infection (25, 26). Pathogens may colonize damaged mucosa, trigger local inflammation, and translocate into the bloodstream (25, 27–29). Viral infections may further alter oropharyngeal mucosa and promote bacterial translocation (30).

### 3.2 The concept of fastidiousness

The term fastidious organism refers to any microorganism with complex or highly specific nutritional requirements, meaning that it will grow only when certain nutrients are incorporated into its medium. Fastidiousness therefore reflects the inherent difficulty encountered in cultivating such organisms by conventional methods. However, the concept of fastidiousness extends well beyond the simple notion of dependency on specific nutrients. It also encompasses the need for diverse chemical signals, some of which rely directly or indirectly on the presence of neighboring species. This interdependence was recognized early on. As early as 1974, Lewis Thomas, in his book *The Lives of a Cell* (31), observed that the vast majority of microbes on Earth cannot be cultivated in isolation because they live in dense, interdependent communities, feeding and supporting each other through complex chemical signals. This vision already acknowledged the impossibility of detecting certain microorganisms using classical culture-based techniques.

It now appears evident that many of the culture-negative OAIs described in earlier series were very likely attributable to fastidious organisms. While a substantial proportion of such cases can be explained by *K. kingae*, additional fastidious pathogens almost certainly contribute and will be revealed as current molecular techniques continue to evolve.

### 3.3 Fastidious and atypical organisms implicated in pediatric OAIs

Beyond *K. kingae*, a growing body of case reports and small series has identified a diverse array of fastidious and atypical microorganisms in pediatric OAIs, many of which were identified only through extended culture techniques or molecular diagnostics. These organisms include HACEK group members, anaerobes, slow-growing Gram-negative bacilli, and atypical bacteria, all characterized by low or negligible culture yield with conventional methods.

*Bartonella henselae*, the agent of cat-scratch disease, has been increasingly recognized as a cause of subacute osteomyelitis in children and adolescents. Vazquez et al. reported a series of pediatric patients presenting with atypical OAI features, reinforcing the need for systematic serological and PCR testing in the appropriate epidemiological context (16). Similarly, *Moraxella lacunata* was identified as the causative agent of subacute osteomyelitis in a child, highlighting that even unusual members of the oropharyngeal microbiota can produce bone infection (17). Among the HACEK group, *Haemophilus parainfluenzae* and *Haemophilus aphrophilus* have been reported in cases of chronic osteomyelitis and Brodie's abscess, often presenting with an indolent course and negative routine cultures (32, 33). *Aggregatibacter actinomycetemcomitans*, another HACEK member, has been implicated in osteomyelitis and epidural abscess in both children and adults, demonstrating that even slow-growing Gram-negative organisms must be considered in the differential diagnosis of culture-negative OAI (22, 34).

Anaerobic bacteria have also been implicated in unusual presentations. *Fusobacterium nucleatum*, a Gram-negative anaerobe normally resident in the oropharyngeal flora, has been identified as the causative pathogen in subacute osteomyelitis and pelvic OAI with Brodie's abscess in children (20, 35). These cases are diagnostically challenging, as anaerobic culture requires specific conditions and results are frequently delayed or negative. *Morganella morganii*, typically considered an enteric organism, was reported as the causative agent of a Brodie's abscess of the pediatric talus, a presentation highlighting the expanding bacteriologic spectrum of subacute OAI (36).

*Staphylococcus caprae*, a coagulase-negative staphylococcus rarely implicated in bone infection, was reported in subacute osteomyelitis in a teenager, with identification requiring extended culture and molecular confirmation (18). Acetabular osteomyelitis in children, a rare and diagnostically challenging presentation, has been managed via hip arthroscopy, with pathogen identification remaining elusive in many cases (37).

Table 2 summarizes the key fastidious and atypical organisms implicated in pediatric OAIs, their clinical presentations, detection methods, and culture yields. HACEK: *Haemophilus*, *Aggregatibacter*, *Cardiobacterium*, *Eikenella*, *Kingella*spp. OAI: osteoarticular infection. PCR: polymerase chain reaction. 16S rRNA: 16S ribosomal RNA sequencing.

**Table 2:** Fastidious and atypical organisms implicated in pediatric osteoarticular infections.

Organism	Age group	Clinical presentation	Detection method	Culture yield
*K. kingae*	< 4 years	Septic arthritis, osteomyelitis, spondylodiscitis; subacute onset	PCR (throat swab/joint fluid), 16S rRNA	Low (< 40%)
Other HACEK organisms (*Haemophilus*, *Aggregatibacter*, *Cardiobacterium*, *Eikenella*, *Kingella* spp.)	All ages	Indolent osteomyelitis, endocarditis-related OAI	Extended culture, PCR, 16S rRNA	Very low
*Abiotrophia*/*Granulicatella*	Any age	Culture-negative OAI, endocarditis-related bone infection	Molecular methods, 16S rRNA sequencing	Very low
*Mycoplasma pneumoniae*	Adolescents	Atypical pneumonia with septic arthritis	PCR, serology	Not applicable
*Bartonella henselae*	Children and adolescents	Subacute osteomyelitis, cat-scratch disease with OAI	PCR, serology	Very low
*Moraxella lacunata*	Children	Subacute osteomyelitis, Brodie-like presentation	Extended culture, PCR	Very low
*Haemophilus parainfluenzae*/*aphrophilus*	Any age	Chronic osteomyelitis, Brodie's abscess	Culture (slow-growing), PCR	Low
*Fusobacterium nucleatum*	Children	Subacute osteomyelitis, pelvic OAI with Brodie's abscess	Anaerobic culture, 16S rRNA	Very low
*Staphylococcus caprae*	Teenagers	Subacute osteomyelitis	Extended culture, molecular methods	Low
*Aggregatibacter actinomycetemcomitans*	Children and adults	Osteomyelitis, epidural abscess	Extended culture, PCR	Very low
*Morganella morganii*	Children	Brodie's abscess (talus)	Culture (uncommon setting)	Variable

## 4 SAHO and culture-negative infections: clinical parallels

When examining the different forms of OAIs, one can reasonably draw an analogy between culture-negative infections and subacute osteomyelitis. They may even be interpreted as two expressions of the same underlying problem. Subacute hematogenous osteomyelitis is defined as a bone infection characterized by localized pain, minimal or absent systemic manifestations, inconclusive laboratory findings, negative blood cultures, but positive radiologic signs (38–45). According to King and Mayo, any bone infection lasting more than 2 weeks but less than 3 months, without acute clinical features, may be referred to as subacute osteomyelitis (39). This relatively broad definition encompasses several entities whose common denominator is a diagnostic delay that can result in lytic lesions.

SAHO likely reflects a particular host-pathogen interaction, which may arise from increased host resistance, reduced virulence of the causative organism, or prior antibiotic exposure (40, 45–47). Standard bone culture techniques usually fail to identify the pathogen, with yields below 25% in most published series. However, as shown in Table 1, the introduction of PCR-based assays has substantially improved pathogen identification in SAHO, with rates exceeding 60% in recent cohorts.

The most comprehensive evidence to date on this topic comes from the Geneva cohort study by Paris et al. (2026), which retrospectively analyzed 107 consecutive SAHO cases managed between 2000 and 2025 (19). This 25-year single-center series demonstrated that classic culture methods identified a pathogen in only 17.8% of patients. The introduction of bone aspirate PCR between 2007 and 2025 increased the yield substantially: PCR on bone aspirates was positive in 78.8% of tested cases, and broad-range 16S PCR added further identifications. Overall, a pathogen was identified in 44.9% of all 107 patients when all microbiological methods were combined. Strikingly, an additional 20 children tested positive for *K. kingae* DNA on oropharyngeal throat swabs alone, despite having undergone no invasive sampling, reinforcing the value of this non-invasive strategy and lending direct clinical support to the conceptual link between oropharyngeal microbiota and SAHO pathogenesis. More than 55% of cases in this series remained without a confirmed pathogen even after comprehensive molecular investigation, pointing to the persistent diagnostic gap and the need for further characterization of fastidious organisms.

The definition of SAHO aligns almost perfectly with the characteristics of culture-negative OAIs, yet this parallel was not formally established in earlier literature. Both conditions share a tendency to remain undiagnosed with conventional culture, a probable contribution from fastidious organisms, a diagnostic delay resulting in more advanced radiological findings, and a clinical course that may be modified by host immunity, pathogen virulence, or prior antibiotic exposure. It is therefore reasonable to consider these scenarios as clinically related, differing primarily by diagnostic delay, with lytic lesions such as Brodie's abscess representing the most severe manifestations of prolonged undiagnosed infection.

## 5 Diagnostic advances (PCR, NGS, mNGS)

### 5.1 Nucleic acid amplification tests

Nucleic acid amplification tests (NAATs), and particularly qPCR assays targeting *K. kingae* and other common OAI pathogens, have substantially improved diagnostic yield in pediatric OAI (12, 13). Species-specific PCR applied to joint fluid, throat swabs, or bone aspirates allows identification of fastidious organisms that would otherwise escape conventional culture. The gain in sensitivity ranges from 20 to 40 percentage points compared to culture alone (Table 1). In the context of SAHO specifically, Paris et al. (2026) demonstrated that bone aspirate PCR was positive in 78.8% of tested cases, while the broader strategy of combining all available molecular methods reached 44.9% overall pathogen identification in a 107-patient cohort over 25 years (19). Oropharyngeal throat swab PCR for *K. kingae* proved valuable as a complementary non-invasive tool, identifying the pathogen in 20 patients who had no positive result from any invasive investigation.

Magnetic resonance imaging (MRI) plays an increasingly prominent role in suspected OAIs by detecting joint effusions and identifying abscesses, whether metaphyseal, transphyseal, periosteal, paraosteal, or within soft tissues, that can be targeted for aspiration to secure a definitive diagnosis or for surgical intervention (48, 49). Combined with molecular diagnostics, MRI-guided sampling may significantly improve pathogen identification rates in both acute and subacute presentations.

### 5.2 Next-generation sequencing (NGS)

Next-generation sequencing (NGS) will likely become indispensable in microbiology, replacing conventional pathogen characterization based on staining, morphology, and metabolic traits with genomic definition. As the most frequently implicated pathogens in OAIs are recognized, NGS will probably evolve from broad parallel sequencing toward targeted amplification of specific genomic regions of interest (18).

The implementation of metagenomic NGS (mNGS) in routine clinical practice is constrained by logistical and technical factors. The technique requires specialized laboratory infrastructure, robust computational pipelines, and skilled personnel to ensure quality at each step of the analytical process. The lack of standardization in sample preparation, sequencing platforms, bioinformatics pipelines, and reporting thresholds complicates inter-laboratory reproducibility. Turnaround times remain variable, and the high cost of sequencing raises concerns regarding cost-effectiveness, particularly in resource-limited settings. Regulatory frameworks and quality assurance standards for clinical application are still evolving, and widespread adoption will require clearer guidelines to ensure reliable and safe use in clinical microbiology laboratories.

For the moment, mNGS can only be applied to specific situations, and it is other nucleic acid amplification tests that help define unexpected pathogens in routine practice. Further validation, standardization, and cost-effectiveness analyses are required before NGS becomes the cornerstone of OAI diagnostic management.

### 5.3 Plasma-based metagenomic NGS and liquid biopsy

Plasma-based metagenomic NGS (mNGS) may soon enable detection not only of pathogens circulating in the bloodstream but also of those responsible for localized infections (15, 50, 51). This liquid biopsy represents a promising diagnostic approach, capable of identifying the full spectrum of OAI pathogens without requiring invasive procedures. Recent work by Ahmed et al. (2025) demonstrated the feasibility of plasma mNGS for the detection of *K. kingae* in pediatric spinal infections (15), reinforcing the potential of this technology in challenging clinical scenarios.

## 6 Clinical implications and future directions

The recognition of a broadening etiologic spectrum, from *K. kingae* to HACEK organisms, anaerobes, *Bartonella henselae*, *Fusobacterium* species, and other fastidious bacteria, has direct implications for antibiotic selection and duration. Empirical therapy should be reviewed in light of updated epidemiological knowledge, and clinical guidelines should incorporate indications for broadening microbiological investigation when conventional cultures are negative.

The integration of oropharyngeal sampling (throat swabs) with molecular diagnostics represents a promising non-invasive strategy, particularly for young children in whom joint or bone sampling may carry procedural risks. Future research should focus on validating multiplex PCR panels targeting the most common fastidious organisms, including the atypical pathogens described in this review.

By allowing rapid OAI detection through MRI and improving pathogen recognition through molecular diagnostics, one can legitimately anticipate that the two entities, culture-negative infections and subacute osteomyelitis, will progressively disappear from the diagnostic landscape. As the most frequently implicated pathogens in OAIs are recognized, NGS will probably evolve from broad parallel sequencing toward targeted amplification of specific genomic regions of interest, further refining the taxonomy of pediatric OAIs and enabling truly personalized antimicrobial therapy.

## 7 Conclusion

Pediatric osteoarticular infections (OAIs) continue to pose a major diagnostic and therapeutic challenge, particularly when conventional cultures fail to identify the causative organism. For decades, culture-negative cases were regarded as enigmas, yet accumulating evidence demonstrates that many of these infections are attributable to fastidious organisms, with *Kingella kingae* representing the paradigm. Advances in molecular diagnostics, especially species-specific PCR and next-generation sequencing, have reshaped our understanding of the true bacteriologic landscape. At the same time, recognition of the oropharyngeal microbiota as a principal reservoir of OAI pathogens has provided new insights into disease pathogenesis and highlighted the importance of host-microbe interactions.

The growing recognition of a diverse range of fastidious and atypical pathogens, including *Bartonella henselae*, HACEK organisms, *Fusobacterium nucleatum*, *Moraxella lacunata*, and others, highlights that the etiologic spectrum of pediatric OAI is far broader than previously appreciated. These organisms share a common thread: they reside in the oropharyngeal microbiota, are poorly detected by conventional culture, and may produce subacute or indolent presentations that further delay diagnosis.

The parallels between culture-negative OAIs and SAHO are striking, suggesting that both may represent clinical expressions of similar microbiological and host factors. The integration of sensitive imaging modalities, such as MRI, with advanced molecular tools now permits earlier diagnosis, improved pathogen identification, and more rational treatment strategies. Looking forward, liquid biopsy approaches including plasma-based metagenomic sequencing hold the potential to further diminish the category of culture-negative infections.

Ultimately, as diagnostic technologies evolve, the very concept of culture-negative pediatric OAIs may gradually disappear, replaced by a more precise, microbiologically informed taxonomy. This transition will not only refine epidemiological knowledge but also enhance patient care by enabling truly personalized antimicrobial therapy.
